# Elevated Expression of Fractalkine (CX3CL1) and Fractalkine Receptor (CX3CR1) in the Dorsal Root Ganglia and Spinal Cord in Experimental Autoimmune Encephalomyelitis: Implications in Multiple Sclerosis-Induced Neuropathic Pain

**DOI:** 10.1155/2013/480702

**Published:** 2013-09-24

**Authors:** Wenjun Zhu, Crystal Acosta, Brian MacNeil, Claudia Cortes, Howard Intrater, Yuewen Gong, Mike Namaka

**Affiliations:** ^1^Faculty of Pharmacy, University of Manitoba, Apotex Center 750, McDermot Avenue, Winnipeg, MB, Canada R3E 0T5; ^2^Faculty of Medicine, University of Manitoba, Winnipeg, MB, Canada R3E 3P5

## Abstract

Multiple sclerosis (MS) is a central nervous system (CNS) disease resulting from a targeted autoimmune-mediated attack on myelin proteins in the CNS. The release of Th1 inflammatory mediators in the CNS activates macrophages, antibodies, and microglia resulting in myelin damage and the induction of neuropathic pain (NPP). Molecular signaling through fractalkine (CX3CL1), a nociceptive chemokine, via its receptor (CX3CR1) is thought to be associated with MS-induced NPP. An experimental autoimmune encephalomyelitis (EAE) model of MS was utilized to assess time dependent gene and protein expression changes of CX3CL1 and CX3CR1. Results revealed significant increases in mRNA and the protein expression of CX3CL1 and CX3CR1 in the dorsal root ganglia (DRG) and spinal cord (SC) 12 days after EAE induction compared to controls. This increased expression correlated with behavioural thermal sensory abnormalities consistent with NPP. Furthermore, this increased expression correlated with the peak neurological disability caused by EAE induction. This is the first study to identify CX3CL1 signaling through CX3CR1 via the DRG /SC anatomical connection that represents a critical pathway involved in NPP induction in an EAE model of MS.

## 1. Introduction

Multiple sclerosis (MS) is a chronic inflammatory autoimmune disease of the central nervous system (CNS) which is characterized by inflammation and subsequent demyelination of brain and spinal cord (SC) [1, 2]. Although the exact pathophysiology of MS is still unknown, it is associated with CNS infiltration of activated inflammatory Th1 cells resulting in axonal myelin damage and subsequent neuronal destruction. The targeted immune mediated destruction of CNS myelin results in a variety of neurological deficits that include but are not limited to ataxia, cognitive dysfunction, weakness, fatigue, motor deficits, and sensory abnormalities such as neuropathic pain (NPP) [1, 3–5].

Chemokines are a family of small cytokines that function as key mediators which control the response of leukocytes in areas of inflammation. They also act as chemotactic cues for leukocytes via interactions with their G-protein coupled, cell membrane-spanning receptors. Currently, fifty chemokines have been identified, which have been divided into four subgroups of chemokines: XC, CC, CXC, and CX3C [6]. Synthesis of chemokines occurs rapidly within infected or damaged tissues. They are thought to drive chronic neuroinflammatory processes in order to attract appropriate cell populations to combat invading organisms and repair damaged CNS tissues [6]. Recent studies aimed at using chemokine antagonists support the importance of chemokines in pain induction, as blocking their molecular signaling has been suggested to ameliorate neurological deficits such as NPP in inflammatory autoimmune disorders such as MS [7, 8].

CX3CL1 (fractalkine) is the only member of the fourth class of chemokines, with a CX3C motif in the mucin-like domain [9, 10]. It is unique in that it is tethered to a cell membrane and is cleaved after an excitotoxic stimulus to produce a soluble, diffusible protein [11]. CX3CL1 is usually expressed in the normal rodent CNS tissue by different neuronal cell subtypes [12]. In addition, it is also expressed in monocytes, natural killer (NK) cells, and smooth muscle cells [13]. Recent evidence has shown that CX3CL1 and its receptor CX3CR1 are known to be involved in the pathogenesis of other clinical diseases such as rheumatoid arthritis, chronic pancreatitis, and NPP [14–17] through their ability to regulate neuronal-microglial communication [18]. In the CNS, CX3CL1 is highly expressed by neurons while CX3CR1 is only expressed by microglia [12, 19]. Specifically, studies have shown that SC microglia expression of CX3CR1 significantly increases in animal models of NPP relative to normal baseline levels of naive controls [13, 20, 21].

Several studies show that induction of NPP results in the synthesis and release of CX3CL1 in the sensory neurons of the dorsal root ganglion (DRG) [21, 22]. Furthermore, this increase is accompanied by the upregulation of CX3CR1 in the SC microglia which correlates with the onset of NPP [21]. The most likely source of CX3CR1 in the SC of animals with NPP is resident microglia which is known to upregulate CX3CR1 in response to injury [13, 21]. However, activated Th1-cells, transmigrating across the blood brain barrier, may also be an additional source of increased CX3CR1-immunoreactivity detected in the SC during NPP [23]. 

Further evidence in support of the nociceptive role of CX3CL1 in NPP development comes from a study using intrathecal injections of CX3CL1 [22]. The results of this study showed that acute intrathecal injection of CX3CL1 resulted in the development of thermal hyperalgesia and mechanical allodynia in adult rats [24], while the administration of neutralizing antibodies against CX3CR1 attenuated the allodynia and hyperalgesia. Taken together, these results directly link the molecular signaling of CX3CL1 through CX3CR1 to the induction of pain [24, 25]. However, in a spared nerve injury model performed in CX3CR1 knockout mice, researchers showed increased allodynia thereby suggesting an alternative nociceptive signaling pathway for CX3CL1 besides that which was solely elicited through CX3CR1 [22].

We *hypothesize* that the molecular signaling of CX3CL1 through its receptor CX3CR1 via the anatomical DRG/SC connection represents a critical pathway involved in the induction of MS-induced NPP. 

In order to confirm the role of CX3CL1 and CX3CR1 in MS-induced NPP, we assessed the gene and protein expression of CX3CL1 and CX3CR1 in a rat EAE model induced by myelin basic protein (MBP) [26]. Our study shows significant increases in the DRG and SC of both CX3CL1 and its receptor CX3CR1. Our results also confirmed that the increased CX3CL1 and CX3CR1 levels correlate with the progression of behavioral sensory abnormalities which is consistent with that of MS-induced NPP. Moreover, we also show a detailed immunohistochemical (IHC) analysis of the cellular distribution of CX3CL1 and CX3CR1 in the EAE SC to identify the cellular sources that contribute to the expression of CX3CL1/CX3CR1. This study confirms the involvement of CX3CL1 signaling through CX3CR1 the induction of in MS-induced NPP. 

## 2. Materials and Methods

### 2.1. EAE Induction

Adolescent female Lewis rats 6–8 weeks of age, weighing 135–150 g (Charles River, Montreal, QC, Canada) were induced to a state of EAE using MBP in accordance with in-house methods previously published [26, 27]. Briefly, rats were maintained at 22°C in a room with automatic light/dark cycles of 12/12 hours. The rats were randomly assigned to three experimental groups: naïve control (NC), active control (AC), and EAE. There were 5 predetermined time points for sacrifice identified at 3, 6, 9, 12, and 15 days postinoculation (DPI) in the AC and EAE groups. For example, EAE animals euthanized at day 3 would be referred to as EAE3 versus AC control animals euthanized at day 3 which would be referred to as AC3. All animal experiments in the present study were conducted according to protocols approved (#10-024/1/2) by the University of Manitoba Animal Protocol Management and Review Committee in full compliance with the Canadian Council on Animal Care. Neurological disability is scored according to the criteria outlined in Table 1. 

### 2.2. Tissue Harvesting for Cryosectioning

For IHC analysis of protein expression, animals were perfusion fixed with 4% paraformaldehyde as previously described [26, 27]. Spinal columns were dissected and decalcified for subsequent cryostat sectioning (10 *μ*m sections) according to previously described and published in-house protocols [28]. The tissue was collected at the various time-dependent stages of disease progression. SC and DRG were collected at days 3, 6, 9, 12, and 15 after inoculations. 

### 2.3. Gene/Protein Analysis

DRG and SC tissues were harvested for gene and protein expression analysis of CX3CL1 and its receptor CX3CR1, as previously described [26, 27]. The freshly harvested tissue was extracted and stored in RNA later stabilization reagent (Qiagen, cat. no. 76106, Washington, DC, USA) until processed. Total RNA and protein were extracted using commercially available kits (All Prep, Qiagen) as described previously [26, 27]. 

### 2.4. Thermal Sensory Testing

Withdrawal latencies to a radiant heat stimulus [29] were assessed for each rat using a Model 336G Plantar/Tail Stimulator Analgesia Meter (IITC Life Sciences, Woodland Hills, CA, USA) according to previously published in-house methods [5]. The time required to move the paw or tail from the heat source was recorded as the withdrawal latency. Rats were habituated to the testing apparatus for 30 minutes, 2 days prior to any testing and for 10 minutes prior to testing on each test day. A standardized 60% light output intensity setting was used for thermal testing. Each experimental group was tested every day after inoculation. Region specific withdrawal responses consisted of licking the paws and flicking the tail in response to the heat stimulus. Withdrawal latencies were recorded in seconds with a maximum of 20 second cut-off point programmed into the timer to prevent tissue damage. Based on our previously published methodology, withdrawal latency was recorded three separate times (seconds) for each paw and tail and average withdrawal latency was calculated. These latencies were then normalized to baseline values and presented as percentages [5, 26]. 

### 2.5. Mechanical Allodynia

To quantify mechanical allodynia, rats were placed in lucite cubicles over top of a metal mesh floor and mechanical stimuli were applied to each hind paw with a 1.0 mm von Frey filament attached to a digitized strain gauge [30]. The maximum force generated before withdrawal is recorded for each hind paw over three trials and averaged. 

### 2.6. IHC Staining

IHC was conducted on 10 *μ*m cryostat sections to detect cellular location of the protein expression according to previously published in-house methods [26, 27]. Double-labeled IHC analysis was conducted using polyclonal antibodies against the neuronal markers NeuN (1 : 100; Chemicon, Billerica, MA, USA), astrocytes marker glial fibrillary acidic protein (GFAP) (1 : 100; Santa Cruz, CA, USA), and microglia marker CD68 (ED1) (1 : 100, Santa Cruz, CA, USA) and were conducted in conjunction with the polyclonal antibody for CX3CL1 (1 : 100; eBioscience, San Diego, CA, USA) and CX3CR1 (1 : 100; eBioscience, San Diego, CA, USA). Secondary antibodies were goat anti-mouse FITC (1 : 100, Jackson, West Grove, PA, USA) and goat anti-rabbit TRITC (1 : 100; Jackson, West Grove, PA, USA). The slides were imaged using the Nikon DS-US camera, and images were captured at the same exposure times and colorized in Image-Pro Plus 6.2. Image sizing, black background balancing, and final collation for publication were performed using Adobe Creative Suite 2 v9.0.2 (Adobe Systems Inc., San Jose, CA, USA). No image manipulations were performed other than those described.

### 2.7. Real Time Reverse Transcription Polymerase Chain Reaction (Real Time RT-PCR)

Real time RT-PCR was conducted on DRG and SC as per previously published in-house methods [26, 27]. The PCR reaction was performed using a Light-Cycler-DNA master SYBR green 1 kit following manufacturers protocols (Bio-Rad, Hercules, CA, USA). CX3CL1 primers were *forward*: 5′-gaattcctggcgggtcagcacctcggcata-3′; *reverse*: 5′-aagcttttacagggcagcggtctggtggt-3′ at an annealing temperature of 60°C. CX3CR1 primers were *forward*: 5′-agctgctcaggacctcaccat-3′; *reverse*: 5′-gttgtggaggccctcatggctgat-3′ at an annealing temperature of 60°C. The cDNAs were amplified by 35 cycles of PCR. Expression levels were normalized to glyceraldehyde-3-phosphate dehydrogenase (GAPDH). GAPDH is an enzyme associated with cell metabolism and is used as a standard housekeeping gene for expression pattern comparisons [31, 32]. GAPDH primers were commercially available from SuperArray. The quantification technique used the standard curve method.

### 2.8. Enzyme Linked Immunosorbent Assay (ELISA)

Total protein was extracted from the SC and DRG as described above, and total protein concentration was assessed using the Bradford assay [33]. The protein concentrations of the samples were adjusted to 10 *μ*g total protein for CX3CL1 and 1 *μ*g total protein for CX3CR1 in the sample volume of 100 *μ*L. Sandwich-style ELISA was performed using the RayBio rat CX3CL1 ELISA kit (RayBio, Norcross, GA, USA) and rat chemokine CX3CR1 ELISA kit (Mybiosource, San Diego, CA, USA) according to the manufacturer's instructions. CX3CL1 and CX3CR1 contents were measured from standard curve runs for each plate (linear range of 0–2000 ng for CX3CL1; 0–10 ng for CX3CR1). Samples from the groups of AC and EAE and the NC rats were determined in each run. Each sample was assayed with 6 replicates per ELISA.

### 2.9. Statistical Analysis

Statistics were performed using GraphPad Prism version 4.03 for Windows, GraphPad Software (San Diego, CA, USA) http://www.graphpad.com/. A statistical analysis for ELISA and Real Time-PCR (RT-PCR) was performed using ANOVA with Tukey's multiple comparison post hoc test. For the behavioral analysis, Student's *t-*test was used to confirm the significance of differences between the means of groups.

## 3. Results

### 3.1. Neurological Disability Scores (NDS)

**Figure 1:**
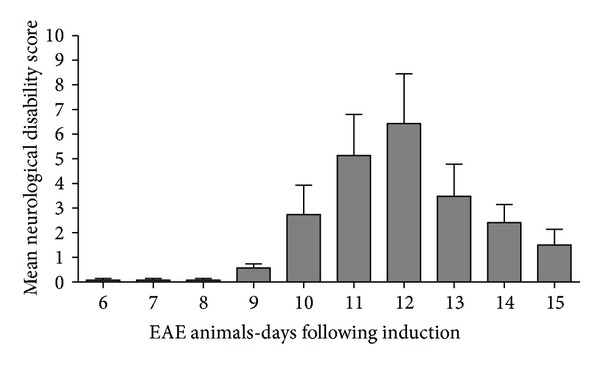
EAE animals Neurological Disability Clinical Score. All animals in the EAE groups were assessed for neurological disability according to a previously described global neurological disability assessment tool [26, 27] detailed in Table 1. Disability scores range from a mean clinical disability score of 0 (no disability) to 15 (maximum disability). The bell shaped distribution outlining peak neurological disability in response to EAE induction occurred at EAE12. Clinical neurological deficits appear at 6 days after antigenic induction. By EAE9 all animals started to display clinical signs of neurological disability (0.57 ± 0.45; mean ± SEM). Neurological disability progressively worsened upon daily assessment until EAE12 (peak disability; 6.42 ± 5.35) and then subsided by EAE15 (1.5 ± 1.41) as the animals entered the remission phase of disease induction, well characterized for this animal model. Errors bars represent SEM.

All animals in the EAE groups were scored for neurological disability according to a previously published in-house global neurological disability assessment tool [26, 27]. Prior to EAE6, none of the animals displayed clinical neurological deficits thereby scoring zero (Figure 1). At EAE6 neurological deficits began to be displayed in some animals in the form of tail weakness. By EAE9 *all animals* started to display clinical signs of neurological disability (0.57 ± 0.45; mean ± standard error of the mean (SEM)). As a result, EAE9 was designated as the *“day of onset of neurological disability.”* Neurological disability progressively worsened upon daily assessment until EAE12 (peak disability; 6.42 ± 5.35) and then subsided by EAE15 (1.5 ± 1.41) as the animals entered the remission/recovery phase of the disease [34] (Figure 1). The control groups (NC and AC) did not show any clinical signs of disability *(data not shown)*. The significant variation in presentation of NDS identified in this animal model of MS represents the characteristic variation of neurological deficits presented in humans with MS [1, 35]. 

### 3.2. Assessment of Thermal Sensory Testing Thermal Hypoalgesia

**Figure 2:**
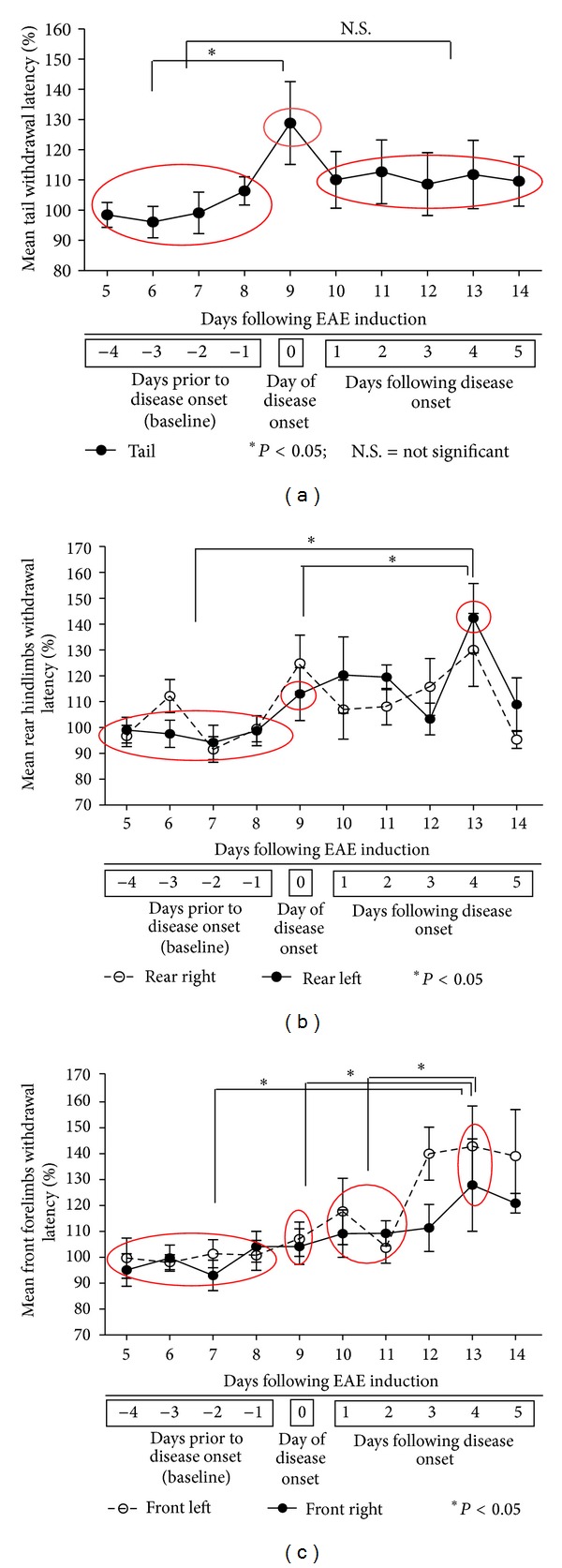
(a) EAE animals: thermal testing for tail. This figure illustrates withdrawal latencies of thermal sensory testing in the tails from EAE animals at different times in the disease progression. Data are aligned on the “*x* axis” to day of onset of neurological disability, where day 0 (=EAE9) is the first day of disease onset (where all EAE animals displayed some form of neurological disability). For example, day 5 is a representative of 5 days following disease onset which equates to EAE14. However, days −4 to −1 (baseline) represent the days prior to the onset of neurological disability that equate to EAE5, EAE6, EAE7, and EAE8 days post-induction. All values were normalized to average baseline withdrawal latencies and displayed as means ± standard error of the mean (SEM). Tail withdrawal latency was significantly elevated at day 0 (day of disease onset = EAE9) compared to the average baseline withdrawal latency, indicative of thermal hypoalgesia in the EAE animals (*P = 0.023 using one sample *t*-test). Following this peak at EAE9, the thermal latency for tail declined and remained stable over the time period of EAE10 to EAE14 days following induction. Withdrawal latencies at baseline were not statistically significantly different from these depicted for days 1, 2, 3, 4 and 5 after disease onset. Errors bars represent SEM. (b, c) EAE animals: thermal testing for hindlimbs and forelimbs. (b, c) illustrate withdrawal latencies of thermal sensory testing in the hindlimb and forelimb of EAE animals at different times in the disease progression. Data are aligned on the “*x* axis” to day of onset of neurological disability, where day 0 (=EAE9) is the first day of disease onset (where all EAE animals displayed some form of neurological disability). For example, day 5 is a representative of 5 days following disease onset which equates to EAE14. However, days −4 to −1 (baseline) represent the days prior to the onset of neurological disability that equate to EAE5, EAE6, EAE7, and EAE8 days following induction. All values were normalized to average baseline withdrawal latencies and displayed as means ± standard error of the mean (SEM). (b) A statistically significant increase (*P < 0.05) of the withdrawal latency characteristic of thermal hypoalgesia was observed in the left hind limb at day 4 following the onset of the disease compared to that of average baseline withdrawal latency (days −1 to −4 inclusive) and to day 0 (onset of neurological disability). (c) Similarly, a statistically significant increase (*P < 0.05) of the withdrawal latency characteristic of thermal hypoalgesia was observed in the right and left forelimbs at day 4 following the onset of the disease compared to average baseline withdrawal latency (days −1 to −4 inclusive); day 0 (onset of neurological disability) and days 1 and 2 after disease onset. Errors bars represent standard error of the mean SEM.

Sensitivity to noxious heat was measured in five specific anatomical domains which included the tail, right and left hind paws, and right and left forepaws. Normalized thermal *tail* withdrawal latencies in EAE animals before and after disease onset are shown in Figure 2(a). Thermal withdrawal latency was measured every day throughout the entire 14-day assessment period. Thermal withdrawal latencies were not measured on EAE15 as that was the day designated for animal sacrifice. The thermal sensory data indicated in Figure 2(a) identifies *day 0* (*onset of neurological disability at EAE9*) as the first day in which all animals started to display clinical neurological deficits. All values were normalized to average baseline withdrawal latencies identified on the “*x* axis” as days −1 to −4 inclusive for comparative analysis at day 0 (onset of neurological disability) and days 1, 2, 3, 4, and 5 after onset of neurological disability which were displayed as means ± SEM. A significantly elevated tail withdrawal latency was identified at day 0 compared to the average baseline withdrawal latency obtained from the withdrawal latencies recorded for the 4 days (−4 to −1) prior to the onset of neurological disability (day 0) (*P < 0.023; using a one sample *t*-test). These findings were consistent with that of thermal hypoalgesia. After this peak at day 0, tail withdrawal latencies decreased over days 2 to 5 when they remained stable and were not statistically significantly different from the average baseline withdrawal latency (days −1 to −4 inclusive). A total of *n* = 9 animals were used for comparative analysis at each of the predetermined experimental time points.

Similarly, a statistically significant increase (*P < 0.05) of the withdrawal latency characteristic of thermal hypoalgesia was observed in the *left hind limb* at day 4 (after disease onset) compared to that of average baseline withdrawal latency (days −1 to −4 inclusive) and to day 0 (onset of neurological disability) as depicted in Figure 2(b). Furthermore, a statistically significant increase (*P < 0.05) of the withdrawal latency characteristic of thermal hypoalgesia was also observed in the *right and left forelimbs* at day 4 (after disease onset) compared to that of average baseline withdrawal latency (days −1 to −4 inclusive), day 0 (onset of neurological disability), and days 1 and 2 after onset of neurological disability as depicted in Figure 2(c). Our results are consistent with the results published by others that have also shown hypoalgesia prior to hyperalgesia in an EAE model of MS [36]. Due to the short duration of our EAE study (15 days), we were only able to demonstrate the early hypoalgesia component of MS-induced NPP using our inflammatory MBP-induced EAE model of MS.

For mechanical allodynia, data were recorded from both hind limbs. An average normalized baseline response was obtained from days −1 to −4 (prior to the onset of neurological disability). Comparative analysis was conducted between average baseline values and those values obtained at day 0 and days 1, 2, 3, 4, and 5 after disease onset. No significant effects for mechanical allodynia were shown using a one sample *t*-test) (*Data not shown*).

### 3.3. CX3CL1 Gene Expression Analysis in the DRG

**Figure 3:**
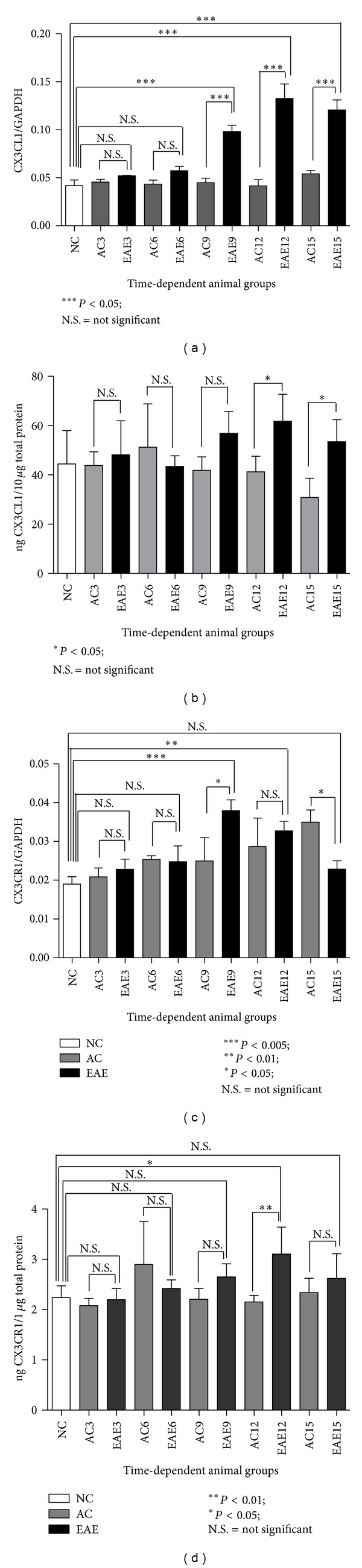
(a–d) Gene and protein expression of CX3CL1 and its receptor CX3CR1 in DRG.

Real time reverse transcription polymerase chain reaction (qRT-PCR) analysis was conducted on DRG isolated from the three experimental groups (EAE, NC, and ACs), at the predetermined experimental time points (Figure 3(a)). The CX3CL1 mRNA expression was assessed in parallel with that of the housekeeping gene (GAPDH). NC animals (white bars) show CX3CL1 mRNA expression at 0.0419 ± 0.0058. AC animals (grey bars) show a similar mRNA expression of CX3CL1 to ACs at all-time points (AC⁡3 = 0.0456 ± 0.0028; AC⁡6 = 0.0434 ± 0.0041; AC⁡9 = 0.0449 ± 0.0047; AC⁡12 = 0.0416 ± 0.0065; and AC⁡15 = 0.0540 ± 0.0035). In comparison, EAE animals (black bars) show a significant increase of CX3CL1 expression in DRG over NC at days 9, 12, and 15 after inoculation (EAE9 = 0.0983 ± 0.0065, *P* < 0.005; EAE12 = 0.1323 ± 0.0154, *P* < 0.005; and EAE15 = 0.1208 ± 0.0102, *P* < 0.005). Furthermore, EAE animals show significant increase in mRNA expression over AC group at days 9, 12, and 15 (*P* < 0.005, *P* < 0.005, and *P* < 0.005). However, there is no significant change of CX3CL1 expression between EAE and NC at days 3 and 6 (EAE3 = 0.0520 ± 0.0006 and EAE6 = 0.0575 ± 0.0045) (ANOVA followed by Tukey's pos thoc test) (Figure 3(a)).

### 3.4. CX3CL1 Protein Expression Analysis in the DRG by ELISA

Total CX3CL1 protein expression is significantly altered in the lumbar dorsal root ganglia of EAE rats starting at day 12 after induction (Figure 3(b)) which directly correlated with the peak of neurological disability scores (Figure 1). Results are given as ng CX3CL1 per 10 *μ*g total protein for each sample. NC animals (white bars) show CX3CL1 protein expression at 44.42 ± 13.58 ng/10 *μ*g of total protein. AC animals (grey bars) show a similar protein expression of CX3CL1 to NC at all-time points (AC⁡3 = 43.85 ± 5.54 ng/10 *μ*g total protein; AC⁡6 = 51.22 ± 17.60 ng/10 *μ*g total protein; AC⁡9 = 41.86 ± 5.43 ng/10 *μ*g total protein; AC⁡12 = 41.25 ± 6.31 ng/10 *μ*g total protein; and AC⁡15 = 30.87 ± 7.77 ng/10 *μ*g total protein). In comparison, EAE animals (black bars) show a significant increase of CX3CL1 expression in DRG over AC at days 12 and 15 (EAE12 = 61.74 ± 10.98 ng/10 *μ*g total protein, *P* < 0.05, and EAE15 = 53.48 ± 8.87 ng/10 *μ*g total protein, *P* < 0.05). However, there is no significant change in CX3CL1 expression between EAE and AC at days 3, 6, and 9 (EAE3 = 48.18 ± 13.79 ng/10 *μ*g total protein; EAE6 = 43.44 ± 4.33 ng/10 *μ*g total protein and EAE9 = 56.82 ± 8.87 ng/10 *μ*g total protein) (ANOVA followed by Tukey's pos thoc test).

### 3.5. CX3CR1 Gene Expression Analysis in the DRG

Total CX3CR1 mRNA expression in the DRG (Figure 3(c)) is significantly elevated at days 9 and 12 following EAE induction, which directly correlates with the onset and peak of neurological disability scoring (Figure 1). Results are shown as a ratio of CX3CR1 mRNA to the housekeeping gene GAPDH. NC animals (white bars) show CX3CR1 mRNA expression at 0.0189 ± 0.0019. AC animals (grey bars) show a similar mRNA expression of CX3CR1 to NC at days 3, 6, 9, and 12 (AC⁡3 = 0.0208 ± 0.0023; AC⁡6 = 0.0254 ± 0.0009; AC⁡9 = 0.0249 ± 0.0060; and AC⁡12 = 0.0287 ± 0.0073) except for the significant change at day 15 (AC⁡15 = 0.0349 ± 0.0032, *P* < 0.01). In comparison, EAE animals (black bars) show a significant increase in CX3CR1 expression in DRG over NC at days 9 and 12 (EAE9 = 0.0379 ± 0.0028, *P* < 0.005, and EAE12 = 0.0327 ± 0.0252, *P* < 0.01). However, there is no significant change in CX3CR1 expression between EAE and NC at days 3, 6, and 15 (EAE3 = 0.0228 ± 0.0026, EAE6 = 0.0248 ± 0.0041 and EAE15 = 0.0228 ± 0.0022). However, EAE animals did show a significant increase in mRNA expression over AC group at days 9 and 15 (*P* < 0.05 and *P* < 0.05 resp.) shown in Figure 3(c) (ANOVA followed by Tukey's pos thoc test). 

### 3.6. CX3CR1 Protein Expression Analysis in the DRG by ELISA

Total CX3CR1 protein expression in the DRG is also altered at day 12 following EAE induction which corresponds to the peak neurological disability scores (Figure 3(d)). Results are given as ng CX3CR1 per 1 *μ*g total protein for each sample. NC (white bars) animals show a baseline level of CX3CR1 in the DRG of 2.24 ± 0.24 ng/1 *μ*g total protein. AC animals (grey bars) show a similar expression level of CX3CR1 compared to that of NC animals at all-time points assessed (AC⁡3 = 2.08 ± 0.15 ng/1 *μ*g total protein; AC⁡6 = 2.90 ± 0.85 ng/1 *μ*g total protein; AC⁡9 = 2.21 ± 0.22 ng/1 *μ*g total protein; AC⁡12 = 2.15 ± 0.13 ng/1 *μ*g total protein, and AC⁡15 = 2.34 ± 0.29 ng/1 *μ*g total protein). In comparison, the EAE (black bars) DRG levels of CX3CR1 protein are significantly increased over NC and AC animals at day 12 (EAE12 = 3.10 ± 0.54 ng/1 *μ*g total protein, *P* < 0.05 and *P* < 0.01); however, EAE animals at days 3, 6, 9 and 15 do not show an increase over baseline (EAE3 = 2.20 ± 0.22 ng/1 *μ*g total protein; EAE6 = 2.42 ± 0.17 ng/1 *μ*g total protein; EAE9 = 2.65 ± 0.26 ng/1 *μ*g total protein, and EAE15 = 2.62 ± 0.49 ng/1 *μ*g total protein) as shown in Figure 3(d) (ANOVA followed by Tukey's po sthoc test). 

### 3.7. CX3CL1 Gene Expression Analysis in the SC

**Figure 4:**
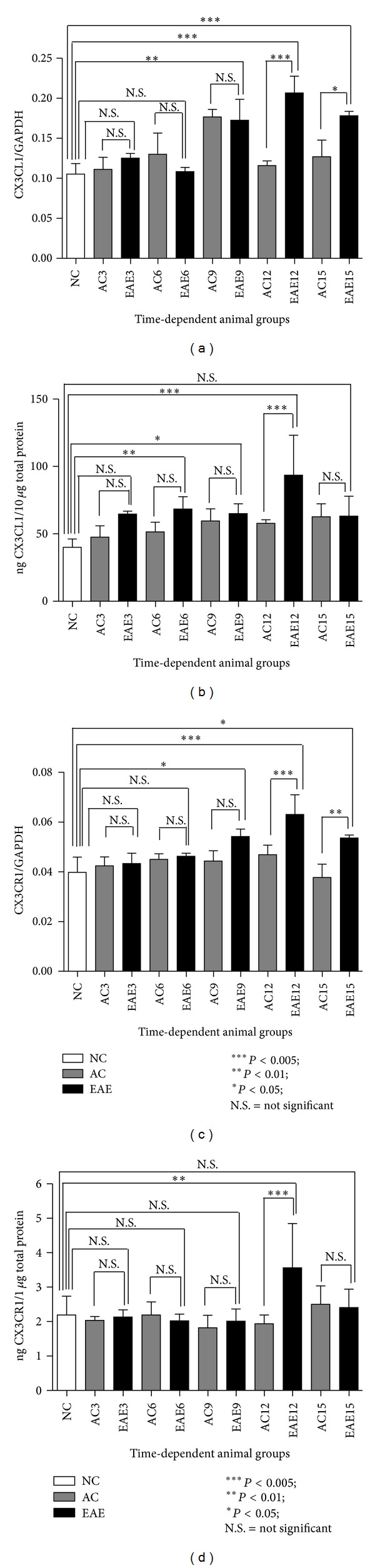
(a–d) Gene and protein expression of CX3CL1 and its receptor CX3CR1 in SC.

CX3CL1 mRNA expression in the SC at different times following EAE induction is shown in Figure 4(a). Results are shown as ratio of CX3CL1 mRNA to GAPDH mRNA. NC animals (white bars) show CX3CL1 mRNA expression at 0.1054 ± 0.0131. AC animals (grey bars) show a similar mRNA expression of CX3CL1 to that of NC animals at all-time points (AC⁡3 = 0.1111 ± 0.0153; AC⁡6 = 0.1301 ± 0.0264; AC⁡9 = 0.1768 ± 0.0092; AC⁡12 = 0.1160 ± 0.0058; and AC⁡15 = 0.1270 ± 0.0209). However, EAE animals (black bars) showed a significant increase of CX3CL1 expression in the SC compared to NC animals at days 9, 12, and 15 (EAE9 = 0.1727 ± 0.026, *P* < 0.01; EAE12 = 0.2067 ± 0.0210, *P* < 0.005; and EAE15 = 0.1783 ± 0.0053, *P* < 0.005). However, no significant change in CX3CL1 expression was identified between EAE and NC animals at days 3 and 6 (EAE3 = 0.1253 ± 0.0059 and EAE6 = 0.1083 ± 0.0052). In addition, EAE animals also showed significant increases in mRNA expression over AC group at days 12 and 15 (*P* < 0.005 and *P* < 0.05, resp.) as shown in Figure 4(a) (ANOVA followed by Tukey's pos thoc test). 

### 3.8. CX3CL1 Protein Expression Analysis in the SC by ELISA

Total CX3CL1 protein expression in the SC is significantly altered at different times following EAE induction as shown in Figure 4(b). Results are given as ng CX3CL1 per 10 *μ*g total protein for each sample. NC animals (white bars) show a baseline level of CX3CL1 in the SC of 40.05 ± 6.09 ng/10 *μ*g total protein. AC animals (grey bars) show a similar expression level of CX3CL1 to that of NC animals at all-time points assessed (AC⁡3 = 47.59 ± 8.33 ng/10 *μ*g total protein; AC⁡6 = 51.45 ± 7.30 ng/10 *μ*g total protein; AC⁡9 = 59.55 ± 8.97 ng/10 *μ*g total protein; AC⁡12 = 57.79 ± 2.62 ng/10 *μ*g total protein; and AC⁡15 = 62.67 ± 9.61 ng/10 *μ*g total protein). In comparison, the EAE (black bars) SC levels of CX3CL1 are significantly increased over NC baseline levels at days 6, 9, and 12 (EAE6 = 68.4 ± 9.16 ng/10 *μ*g total protein, *P* < 0.01; EAE9 = 65.06 ± 7.29 ng/10 *μ*g total protein, *P* < 0.05; and EAE12 = 93.61 ± 29.61 ng/10 *μ*g total protein, *P* < 0.005); however, days 3 and 15 do not show a significant increase over NC baseline levels (EAE3 = 64.74 ± 2.11 ng/10 *μ*g total protein and EAE15 = 63.20 ± 14.76 ng/10 *μ*g total protein). In addition, EAE animals SC levels of CX3CL1 protein are significantly increased over AC animals at day 12 (EAE12 = 93.61 ± 29.61 ng/10 *μ*g total protein, *P* < 0.005), however, at days 3, 6, 9, and 15 do not show a significant increase over AC animals as shown in Figure 4(b) (ANOVA followed by Tukey's pos thoc test). 

### 3.9. CX3CR1 Gene Expression Analysis in the SC

The qRT-PCR results show that CX3CR1 mRNA expression in the SC is not significantly altered at different times following EAE induction as shown in Figure 4(c). NC animals (white bars) show CX3CR1 mRNA expression at 0.0398 ± 0.0061. AC animals (grey bars) at days 3, 6, 9, 12, and 15 (AC⁡3 = 0.0424 ± 0.0036; AC⁡6 = 0.0450 ± 0.0023; AC⁡9 = 0.0443 ± 0.0041; AC⁡12 = 0.0468 ± 0.0039; and AC⁡15 = 0.0377 ± 0.0054) show a similar mRNA expression of CX3CR1 to that of NC animals. In comparison, EAE animals (black bars) at days 9, 12, and 15 (EAE9 = 0.0542 ± 0.0029, *P* < 0.05, EAE12 = 0.0630 ± 0.079, *P* < 0.005; and EAE15 = 0.0536 ± 0.0015) show a significant increase in CX3CR1 expression in SC compared to that of NC animals. However, there is no significant change of CX3CR1 expression in EAE animals at days 3, 6, and 9 (EAE3 = 0.0433 ± 0.0041, EAE6 = 0.0462 ± 0.0120, and EAE9 = 0.0542 ± 0.0029) when compared in that of AC animals at the same time points. Furthermore, EAE animals at days 12 and 15 show significant increase in mRNA expression over AC animals at the same time points (*P* < 0.005 and *P* < 0.05, resp.) as shown in Figure 4(c) (ANOVA followed by Tukey's pos thoc test). 

### 3.10. CX3CR1 Protein Expression Analysis in the SC by ELISA

Total CX3CR1 protein expression in the SC at different time points following EAE induction is shown in Figure 4(d). Results are given as ng CX3CR1 per 1 *μ*g total protein for each sample. NC animals (white bars) show a baseline level of CX3CR1 in the SC of 2.20 ± 0.54 ng/1 *μ*g total protein. AC animals (grey bars) show a similar expression level of CX3CR1 to that of NC animals all at time-points assessed (AC⁡3 = 2.03 ± 0.12 ng/1 *μ*g total protein; AC⁡6 = 2.19 ± 0.38 ng/1 *μ*g total protein; AC⁡9 = 1.82 ± 0.36 ng/1 *μ*g total protein; AC⁡12 = 1.94 ± 0.26 ng/1 *μ*g total protein, and AC⁡15 = 2.50 ± 0.53 ng/1 *μ*g total protein). However; EAE (black bars) SC levels of CX3CR1 protein are significantly increased over NC and AC animals at day 12 (EAE12 = 3.56 ± 1.29 ng/1 *μ*g total protein, *P* < 0.05 and *P* < 0.005), however, days 3, 6, 9, and 15 do not show a significant protein increase over NC and AC baseline protein expression levels (EAE3 = 2.13 ± 0.21 ng/1 *μ*g total protein; EAE6 = 2.02 ± 0.19 ng/1 *μ*g total protein; EAE9 = 2.01 ± 0.36 ng/1 *μ*g total protein, and EAE15 = 2.41 ± 0.53 ng/1 *μ*g total protein) as shown in Figure 4(d) (ANOVA followed by Tukey's pos thoc test). 

### 3.11. IHC Analysis of CX3CL1 Protein Expression in the SC

**Figure 5:**
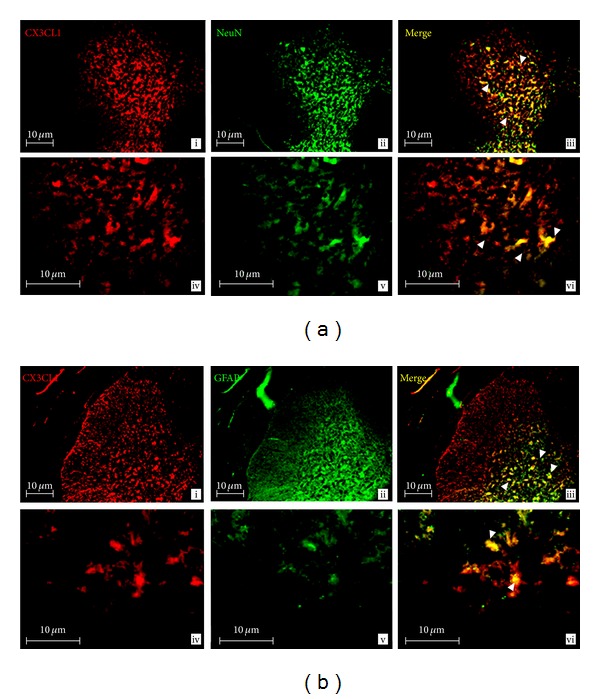
(a, b) CX3CL1 expression in the SC of EAE12 rats. Double-labeled immunofluorescence for CX3CL1 and markers for neurons (NeuN) or astrocytes (GFAP) in the SC of EAE12 rats. (a) CX3CL1 was expressed in neurons in the grey matter of SC. CX3CL1 (panels i and iv: red) in neurons (NeuN: panels ii and v: green). CX3CL1 co-localizes with neurons (panels iii and vi: yellow, arrows). (b) Colocalization between CX3CL1 and astrocytes was observed in the grey matter of SC. CX3CL1 (panels i and iv: red) in astrocytes (GFAP: panels ii and v: green). CX3CL1 colocalizes with astrocytes (panels iii and vi: yellow, arrows). Images were taken at a total magnification of 100x (panels i–iii) and 400x (panels iv–vi) from EAE12 group. Scale bars = 10 *μ*m.

**Figure 6:**
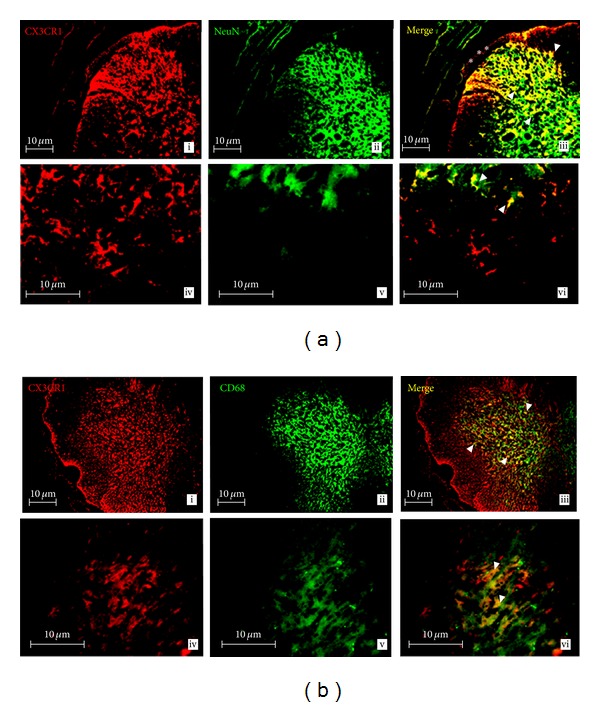
(a, b) CX3CR1 expression in the SC of EAE12 rats. Double-labeled immunofluorescence for CX3CR1 and markers for microglia (CD68) or neurons (NeuN) in the SC of EAE12 rats. (a) Colocalization between CX3CR1 and neurons was observed in the dorsal horn (in asterisk) of SC. CX3CR1 (panels i and iv: red) in neurons (NeuN: panels ii and v: green). CX3CR1 colocalizes with neurons (panels iii and vi: yellow, arrows). (b) CX3CR1 was expressed in microglia in the grey matter of SC. CX3CR1 (panels i and iv: red) in microglia (CD68: panels ii and v: green). CX3CR1 colocalizes with microglia (panels iii and vi: yellow, arrows). Images were taken at a total magnification of 100x (panels i–iii) and 400x (panels iv–vi) from EAE12 group. Scale bars = 10 *μ*m.

Expression of CX3CL1 and its receptor, CX3CR1, at EAE12, show immunoreactivity in the SC grey matter in different cell types (Figures 5 and 6). Specifically, CX3CL1 (red labeling) was found in neurons (NeuN: green labeling) as shown by double labeling of CX3CL1 with NeuN (yellow labeling, white arrow in Figure 5(a)). Likewise, CX3CL1 was expressed in glial cells, as colocalization of CX3CL1 with GFAP (astrocyte marker: green labeling) was observed (yellow labeling, white arrows in Figure 5(b)) in EAE12 rats. 

### 3.12. IHC Analysis of CX3CR1 Protein Expression in the SC

Expression of CX3CR1 (red labeling) in neurons (NeuN: green labeling) was confirmed by colocalization of CX3CR1 with NeuN (yellow labeling, white arrow in Figure 6(a)) which was predominantly concentrated in the dorsal horn of SC (asterisk). As shown by colocalization of CX3CR1 (red labeling) with CD68 (macrophage marker: green labeling), CX3CR1 is expressed in microglia (yellow labeling, white arrow in Figure 6(b)). Images were taken at a total magnification of 100x (top panels) and 400x (bottom panels) from EAE12 rats.

## 4. Discussion

MS is an autoimmune disease whose pathology involves many of the same inflammatory mediators (tumor necrosis factor alpha (TNF*α*)) that are also commonly associated with the development of chronic pain syndromes such as NPP [5, 37]. The EAE rat model of MS-induced NPP is an ideal model to identify the early molecular mechanisms underlying the pathophysiology of NPP because this model characteristically induces immune system mediated inflammation without demyelination [26, 27]. As a result, this NPP model allows for the identification of molecular changes in pain induction from the earliest onset of an initial immune system mediated inflammatory event known to induce NPP prior to any demyelination. 

NPP has been reported as the second worst disease-induced symptom reported to occur in up to 75% of patients with MS [1, 38]. Interestingly, NPP has also been reported to be present in MS patients prior to the time of diagnosis and therefore may be a promising prediagnostic indicator to facilitate the early diagnosis of MS [39]. 

Previous studies have also shown that EAE animals experience NPP as part of their immune system mediated disease progression [36]. CX3CL1 and CX3CR1 are established factors in the modulation of pain perception via a central proalgesic mechanism [40]. In our study, we demonstrated using an EAE model of MS-induced NPP significant changes in CX3CL1 and its receptor CX3CR1 in the DRG and SC. Specifically, our study confirms that CX3CL1 expression is increased in the DRG and SC during the early inflammatory phase of EAE induction prior to the demyelination tissue damage. As a result, this study confirms the importance of the immune system in pain induction prior to any detectable tissue damage or injury. Interestingly, the increased expression of CX3CL1 directly correlates with the behavioral data that confirms thermal hypoalgesia (a sensory abnormality known to occur in NPP prior to hyperalgesia [36]). As a result, our molecular and behavioral findings suggest that CX3CL1 is a nociceptive mediator induced in the early stages of inflammation by the immune system prior to any detection of myelin damage or injury. Henceforth, CX3CL1 is a nociceptive mediator involved in the early induction of immune system mediated MS-induced NPP. 

NPP is a chronic pain syndrome that has been associated with abnormal sensory changes in response to mechanical, chemical, or thermal stimuli. Due to the variability by which NPP presents, it is unlikely to display sensory abnormalities in response to three forms of stimuli at once. Our results are consistent with the literature in this regard, as our EAE animals only displayed sensory abnormalities to thermal rather than mechanical stimuli. Our study confirms the importance of testing for all three forms of sensory stimuli to ensure all subdomains of NPP are properly tested to confirm their presence or absence in the models being tested.

CX3CL1 is the only member of the fourth group of chemokines with the CX3C motif. It exists in two forms: membrane-bound tethered to the cell membrane by a mucin-like stalk and as a soluble protein following cleavage [10]. CX3CL1 is constitutively expressed by neurons in the brain, SC and DRG [20, 21]. Under normal physiological conditions, membrane-bound CX3CL1 is cleaved by ADAM17 (a matrix metalloproteinase formerly known as TNF converting enzyme (TACE)) to release soluble CX3CL1 [41]. In inflammatory states, increased expression of CX3CL1 occurs in neurons and also in astrocytes in the dorsal horn of the SC [13]. Interestingly, peripheral nerve injury results in a decrease in membrane-bound CX3CL1 within DRG neurons [16] but not in the dorsal horn of the SC [13, 21]. The CX3CL1 receptor, CX3CR1, is constitutively expressed in microglia of the brain and SC [13, 19] and is significantly increased as a result of microglial activation [12, 21]. CX3CR1 is known to be critical for the generation of NPP, as mice lacking CX3CR1 do not develop allodynia following peripheral nerve injury [42]. As a result, the results from our research also support the role of CX3CL1 and its receptor CX3CR1 in the induction of MS-induced NPP. Furthermore, our results also support the importance of the key anatomical connection between the DRG and SC as an integral molecular signaling pathway for which CX3CL1 can exert its pathological effects associated with the induction of NPP through its CX3CR1 receptor. Although we used antigenic induction of the immune system to elicit an inflammatory response to trigger the behavioral changes consistent with NPP, other research using noxious electrical stimulation also linked CX3CL1 to the induction, amplification, and maintenance of injury-induced pain [43].

Following inflammation, injured neurons release adenosine-5′-triphosphate (ATP), which binds to the P2X7 receptor on microglia that subsequently causes the release of the protease Cathepsin S [44]. CX3CL1 is bound to the neuronal membrane and is cleaved by the Cathepsin S [45]. Soluble CX3CL1 binds to the CX3CR1 on microglia resulting in the increased synthesis and release of pronociceptive mediators such as IL-6 and nitric oxide [24]. These pro-nociceptive mediators bind to receptors on dorsal horn SC neurons resulting in enhanced hypersensitivity and spontaneous firing that characterize central pain [24] which subsequently creates a positive feedback loop that pathogenically maintains the CX3CL1/CX3CR1 signaling pathway. 

Early studies have shown that CX3CL1 and CX3CR1 play important roles in neuron-glia communication [43]. In our previous published studies [5, 27], we showed significantly the upregulation of the proinflammatory cytokine TNF*α* at the gene and protein levels within the DRG and SC in the EAE model of MS. Recent research has shown that TNF*α* induces CX3CL1 expression in endothelial cells [40]. Furthermore, TNF*α* also has functional implications in the posttranscriptional regulation of CX3CL1 [46, 47]. These findings indicate that TNF*α* is a critical upstream factor that regulates CX3CL1 production. Thus, our previously published studies in this area suggest that upregulation of TNF*α* in the DRG and SC may be a critical early step in regard to the regulation of MS pain induction via CX3CL1/CX3CR1 signaling pathway. However, further studies are required to definitively confirm this molecular signaling link between TNF*α* and CX3CL1. Additional studies are also required to study the effect of TNF*α* on CX3CL1 expression in SC neurons, astrocytes, and microglia. 

Our data shows significant changes in CX3CL1 and its receptor CX3CR1 at both gene and protein levels within the DRG and SC. Interestingly, these changes also correlated with the onset and peak NDS. Henceforth, we conclude that the changes in CX3CL1 and CX3CR1 expression levels are the direct result of activation of the immune response by CNS-myelin-specific antigens such as MBP. Based on our experimental findings, we also propose that CX3CL1 may be involved in the molecular signaling cascade that ultimately contributes to myelin damage and subsequent neurological disability associated with MS. However, further investigation of this concept needs to be conducted before any definitive conclusions can be drawn in this regard as the events identified in an EAE animal model do not always directly reflect those events which occur in humans with MS. Irrespectively, it is our belief that the CX3CL1/CX3CR1 signaling crosstalk between neurons and microglia in the SC may be involved in the underlying pathology associated with MS [43]. Additional research also supports the concept of CX3CL1/CX3CR1 involvement in the pathogenesis of MS. For example, researchers have linked CX3CL1 to the recruitment of NK cells that modify EAE within the CNS [48]. Furthermore, others have demonstrated that blockade of CX3CL1 protected mice against EAE [49]. However, it is still unclear how the synthesis and secretion of CX3CL1 are regulated and which pathways of CX3CL1 signaling in glia cells are utilized to exert these effects. 

Our study demonstrated that, in an inflammatory state, CX3CL1 is expressed in neurons and astrocytes, and its receptor CX3CR1 is expressed predominantly in microglia. A remarkable finding of our study is that, in the EAE model, CX3CL1 and its receptor (CX3CR1) show significant changes in the expression pattern that correlates with the onset of sensory abnormalities, indicating that the activation of glial cells by an inflammatory response leads to increased pain signaling between neurons and microglia. Our finding that CX3CR1 immunoreactivity is localized on microglia and dorsal horn SC neurons is interesting because CX3CR1 is usually expressed on microglia in the CNS [40]. Thus, our findings indicate that neuronal expression of CX3CR1 occurs as a direct result of CNS inflammation. This expression change in CX3CR1 in SC neurons may be a critical mechanism involved in MS-induced NPP. Further, our study demonstrated that CX3CL1 is expressed in neurons, but its receptor CX3CR1 is expressed predominantly in microglia. This finding is in concordance with previously published studies showing that CX3CL1 works as a molecule signaling from neuron to microglia to induce glia activation. SC microglia and astrocytes have been shown to be implicated in various types of NPP such as peripheral nerve injury, bone cancer, and spinal root constriction [25, 50], thereby confirming that glia activation is directly involved in inflammation and NPP. Recent research has shown that administration of glial inhibitors exerts antiallodynic function [51, 52]. In our research, we found that astrocytes also express CX3CL1 in the EAE SC, suggesting that activated astrocytes are also involved in the induction of pain. 

Our data suggests that, during the early inflammatory stage of MS prior to demyelination, CX3CL1 signaling in dorsal horn SC neurons activates the ascending pathways involved in nociceptive transmission. Interestingly, increased serum levels of CX3CL1 have been reported to be seen in MS patients without significant changes in CX3CL1 levels in the cerebral spinal fluid (CSF) [53]. Studies are ongoing to investigate the correlation between serum levels of CX3CL1 in patients with relapsing remitting MS at different stages of their disease (relapse versus stable remission phase). Henceforth, our study suggests that CX3CL1 and/or its receptor CX3CR1 may be easily assessed biomarkers of MS-induced NPP. 

## 5. Conclusion

Previous research on chronic pain predominantly identified drug, injury, or disease induced causes of pain without fully considering the impact of the immune system on the contribution to chronic pain development. Henceforth, current research has now placed a greater emphasis on the early aspects of pain induction by exploring the neuroimmune modulation of the pain response [5]. In our study we used an MBP animal model of MS that depicts immune system activation of inflammation without demyelination. As such, this EAE model represents an ideal model to study the molecular changes that occur at the earliest stages of immune system activation prior to any tissue damage. The ability to target the molecular changes occurring at the earliest phase of pain induction following immune system activation could minimize the involvement of the downstream nociceptive mediators involved in the induction and/or maintenance of chronic pain. Previous research has shown that TNF*α* induces CX3CL1 expression [40] and that it has functional implications in the post-transcriptional regulation of CX3CL1 [46, 47]. Our current findings of CX3CL1 expression correlate closely with our previously published studies on TNF*α*, thereby supporting the concept that TNF*α* is an integral factor associated with the induced production of CX3CL1. Taken together, these studies suggest that the immune mediated upregulation of TNF*α* in the DRG and SC may be a critical upstream signaling pathway that regulates MS pain induction by governing CX3CL1 expression. However, further studies are required to definitively confirm this molecular signaling link between TNF*α* and CX3CL1. Furthermore, our research also showed significant elevations in the expression of CX3CL1 and its receptor CX3CR1 at both the gene and protein levels within the DRG and SC that correlated with the behavioral data suggestive of NPP. As a result, novel therapeutic interventions aimed at blocking CX3CR1 may prove to be beneficial in attenuating sensory abnormalities associated with neuropathies associated with CX3CL1 induction. In addition, our findings confirmed the expression changes in CX3CL1 and CX3CR1 to occur within the DRG and SC. As a result, our research confirms the importance of the key anatomical connection between the DRG and SC via the connecting dorsal roots as being a critical pathway for the upstream nociceptive molecular signaling of CX3CL1/CX3CR1 following neuroimmune activation. Our findings also confirmed that neurons and astrocytes in the SC express CX3CL1 while neurons and microglia express CX3CR1. As a result, our findings are consistent with other researches that support the involvement of microglia activation in the SC in regard to the induction and/or maintenance of chronic NPP [54]. However, additional studies are required to determine the effect of TNF*α* on CX3CL1 expression in SC neurons, astrocytes, and microglia. In summary, our EAE study results suggest that CX3CL1 and its receptor CX3CR1 may be suitable easily assessed biomarkers of MS-induced NPP that could assist clinicians in the diagnosis and early treatment intervention of MS-induced NPP.

## Figures and Tables

**Table 1:** Neurological Disability Clinical Scoring System for EAE animals induced to a state of MS. The total score is the sum of the following individual scores obtained for each of the 6 specified clinical domains. Following induction, each rat was assessed twice daily for clinical signs of EAE as previously described [27] thereby rendering an average daily mean score. EAE animals after disease onset were assessed three times daily thereby rendering an average daily mean score. Daily body weight and hydration status were also measured to assess general animal health and well-being.

Tail	Bladder
0—normal	0—normal
1—partially paralyzed, weakness	1—incontinence
2—completely paralyzed, limp	

Right hindlimb	Left hindlimb

0—normal	0—normal
1—weakness	1—weakness
2—dragging with partial paralysis	2—dragging with partial paralysis
3—complete paralysis	3—complete paralysis

Right forelimb	Left forelimb

0—normal	0—normal
1—weakness	1—weakness
2—dragging, not able to support weight	2—dragging, not able to support weight
3—complete paralysis	3—complete paralysis
